# NVVC/NHJ Durrer prizes 2014

**DOI:** 10.1007/s12471-015-0691-8

**Published:** 2015-04-17

**Authors:** E.E. van der Wall, V.A.W.M. Umans

**Affiliations:** Netherlands Society of Cardiology/Holland Heart House, Moreelsepark 1, 3511 EP Utrecht, The Netherlands; Department of Cardiology, Medical Center Alkmaar, Wilhelminalaan 12, 1815 JD Alkmaar, The Netherlands

**Keywords:** Durrer prizes, Congress Netherlands Society of Cardiology (NVVC), Netherlands Heart Journal

## Abstract

At the annual 2015 Spring Congress of the NVVC, the Durrer prizes were awarded to the authors of two of the best original/review articles published in the year 2014, one paper being more basically oriented and one paper being more clinically oriented. This has been an annual tradition since the year 2006.

For the ninth time in a row, the *Netherlands Heart Journal (NHJ)*, the official journal of the Netherlands Society of Cardiology (NVVC), awarded the Durrer Prizes to the two best *NHJ* articles published in the year 2014. In 2006, it was thought appropriate by the NVVC Board to set up a special publication prize to stimulate the submission of outstanding scientific articles to *NHJ*. Two articles per year are selected, one article with a more basically oriented character and one with a mainly clinical focus [1–4].

The *NHJ* publication prize carries the name of one of the fathers of Dutch Cardiology, Professor Dirk Durrer (1918–1984), head of the Department of Cardiology in the ‘Wilhelmina Gasthuis’, Amsterdam, who performed pioneering work in the field of electrical activation of the heart in the 1960s and 1970s [5, 6]. Furthermore, Dirk Durrer founded the Interuniversity Cardiology Institute of the Netherlands (ICIN)in 1972, of which he was director until 1983.

From a total of 162 articles published in *NHJ* in 2014, we selected as best basically oriented article: *Left cardiac sympathetic denervation in the Netherlands for the treatment of inherited arrhythmia syndromes.* Authors: Olde Nordkamp LR, Driessen AH, Odero A, Blom NA, Koolbergen DR, Schwartz PJ, Wilde AA. Neth Heart J. 2014;22:160–6 [7]. In this article, an overview of the indications and outcomes of left cardiac sympathetic denervation (LCSD) is provided in patients with inherited arrhythmia syndromes in the Academic Medical Centre (AMC), Amsterdam. It was a retrospective study, including 17 patients, of whom 12 with long-QT syndrome patients (71 %) and 5 patients (29 %) with catecholaminergic polymorphic ventricular tachycardia who underwent LCSD in the AMC between 2005 and 2013. LCSD involved ablation of the lower part of the left stellate ganglion and the first four thoracic ganglia. The major finding of the study was that LCSD for inherited arrhythmia syndromes, which is applied on a relatively small scale in the Netherlands, reduced the cardiac event rate in 87 % of the high-risk patients who had therapy-refractory cardiac events, while the rate of major complications was low. Therefore, LCSD seems a viable treatment for patients with inherited arrhythmia syndromes without other options for therapy. This article has been cited, among others, in the *Journal of Translational Medicine* and in *Circulation*.

The best clinical article selected was *Antithrombotic therapy in patients undergoing TAVI: an overview of Dutch hospitals.* Authors: Nijenhuis VJ, Stella PR, Baan J, Brueren BR, de Jaegere PP, den Heijer P, Hofma SH, Kievit P, Slagboom T, van den Heuvel AF, van der Kley F, van Garsse L, van Houwelingen KG, Van’t Hof AW, Ten Berg JM. Neth Heart J. 2014;22:64–9 [8]. In this article, the current antithrombotic treatment strategies in the Netherlands in patients undergoing transcatheter aortic valve implantation (TAVI) were assessed. To that purpose, for every Dutch hospital performing TAVI (*n* = 14) an interventional cardiologist experienced in performing TAVI was interviewed concerning heparin, aspirin, thienopyridine and oral anticoagulation treatment in patients undergoing TAVI. It was shown that the antithrombotic policy for patients undergoing TAVI is rather variable: aspirin was prescribed in all centres, clopidogrel in 13 of the 14 centres whereby the duration of clopidogrel therapy was 3 months in over two-thirds of cases. As a standardised regimen might further reduce haemorrhagic complications, large randomised clinical trials may help to establish the most appropriate approach. This national multicentre study has been cited in, among other journals, the *JAMA Journal of the American Medical Association*.

At the annual spring meeting of the NVVC, held at the Congress Centre ‘De Leeuwenhorst’ in Noordwijkerhout, 9–10 April 2015, the first authors of both articles received an educational grant provided by the NVVC. We would like to congratulate the authors on their awards and thank them for sending their excellent work to *NHJ*. With the Durrer Prizes, we again hope to stimulate young investigators to send their best papers to the *NHJ*.

E.E. van der Wall, Editor-in-Chief, Netherlands Heart Journal

V.A.W.M. Umans, Chairman of the Netherlands Society of Cardiology
